# Supplementary material for: Five colour variants of bright luminescent protein for real-time multicolour bioimaging
Source: Nat Commun. 2016 Dec 14;7:13718. doi: 10.1038/ncomms13718 (PMC5171807; doi:10.1038/ncomms13718)
Supplement: Supplementary Information — Supplementary Figures 1-16, Supplementary Tables 1-3, Supplementary Notes 1-4 and Supplementary References. [file ncomms13718-s1.pdf]

Supplementary Figure 1. Design of linker truncation library between Nluc and either Venus or mNeonGreen

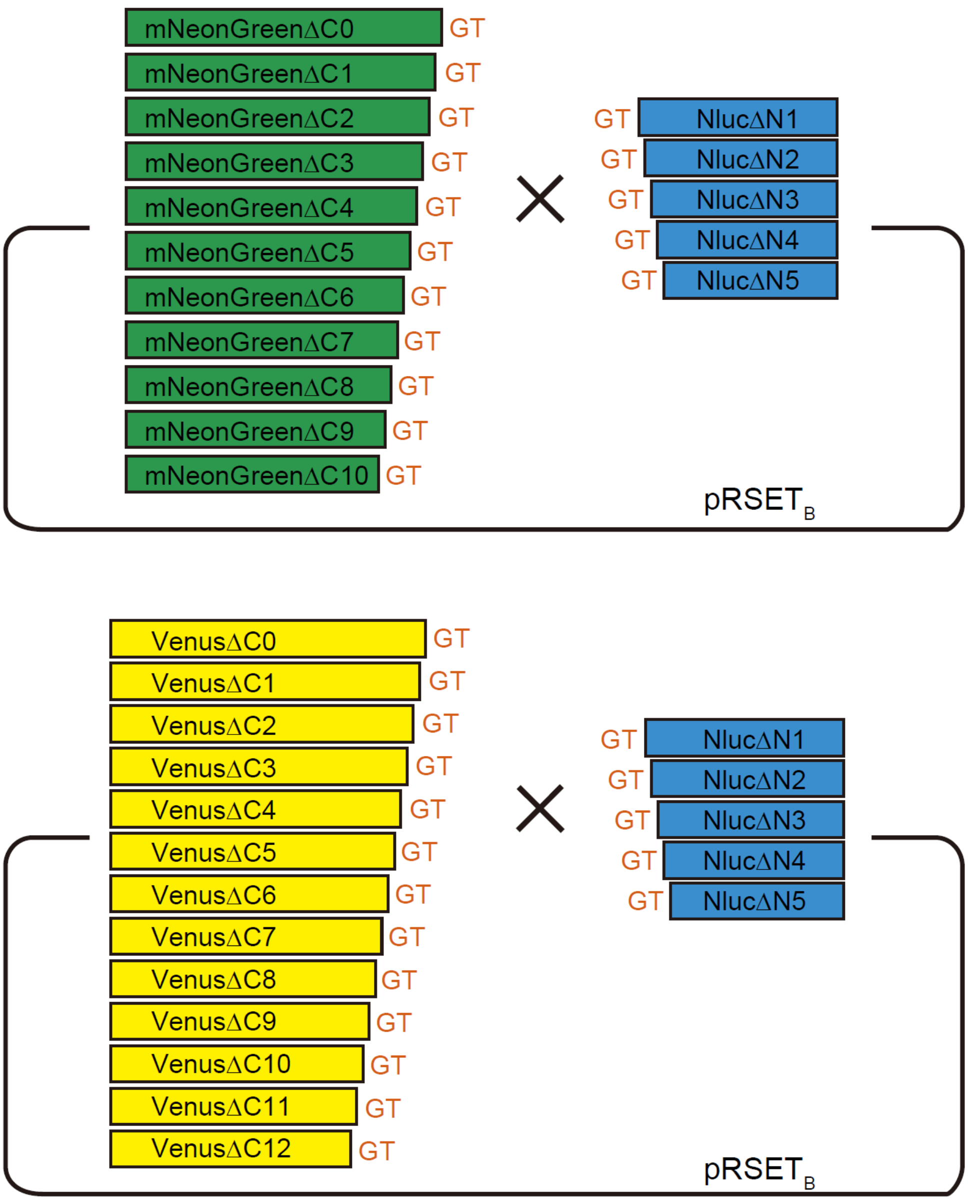

The cDNA of C-terminally deleted FPs (mNeonGreen or Venus) mutants and N-terminally deleted Nluc mutants with two residues GT (highlighted in orange color), derived from the recognition sequence of *Kpn*I (ggtacc), were mixed together, and subcloned in-frame into the *BamH*I/*EcoR*I sites of pRSET$_B$. We generated and screened 55 and 65 linker combinations of mNeonGreen-Nluc and Venus-Nluc pairs, respectively.

Supplementary Figure 2. Emission spectra of linker truncation variants of mNeonGreen-Nluc and Venus-Nluc

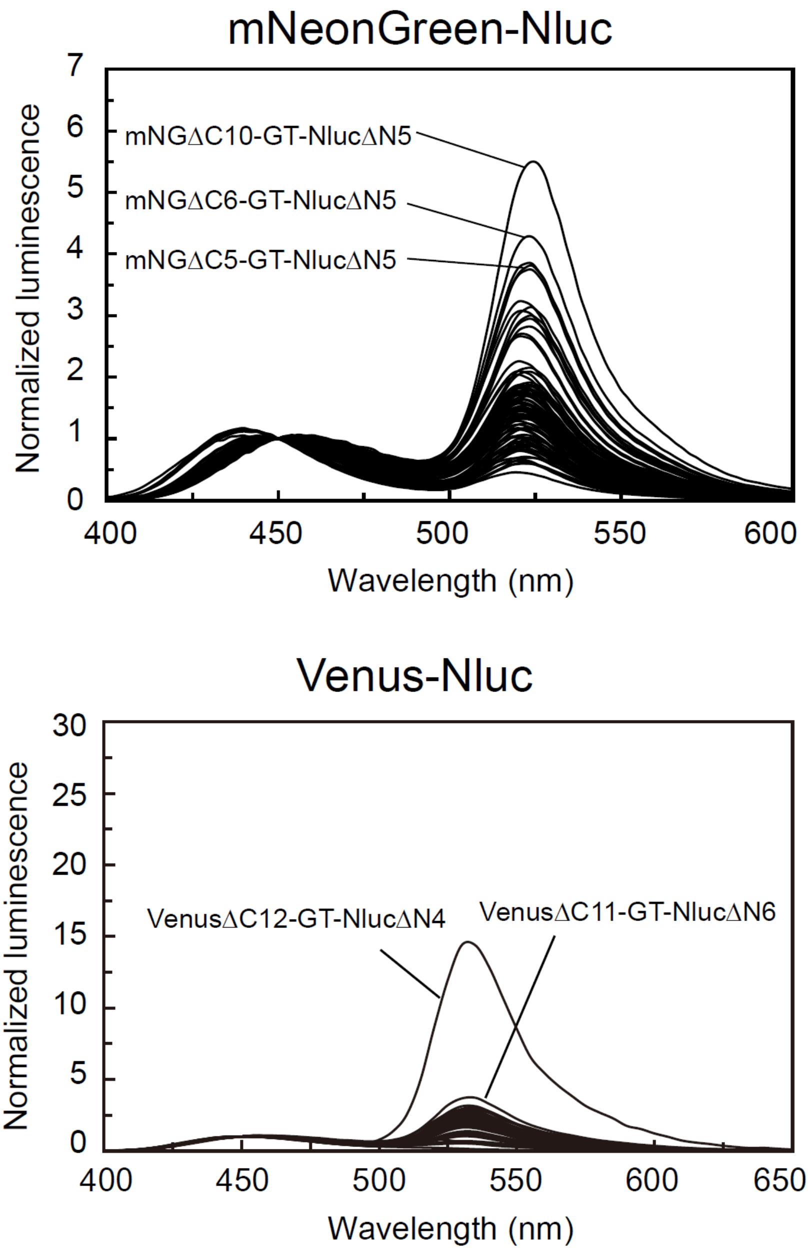

Luminescence spectra of *E. coli* suspension colonies that showed bright luminescence signal on an agar plate at first screening. The spectra were normalized to the peak of Nluc emission intensity. Spectra were measured by micro-plate reader (SH-9000; Corona Electric). Among them, mNeonGreenΔC10-GT-NlucΔN5 and VenusΔC12-GT-NlucΔN4 exhibited high FRET efficiency.

Supplementary Figure 3. Design of randomized linker library in mNeonGreen-Nluc and Venus-Nluc fusion protein

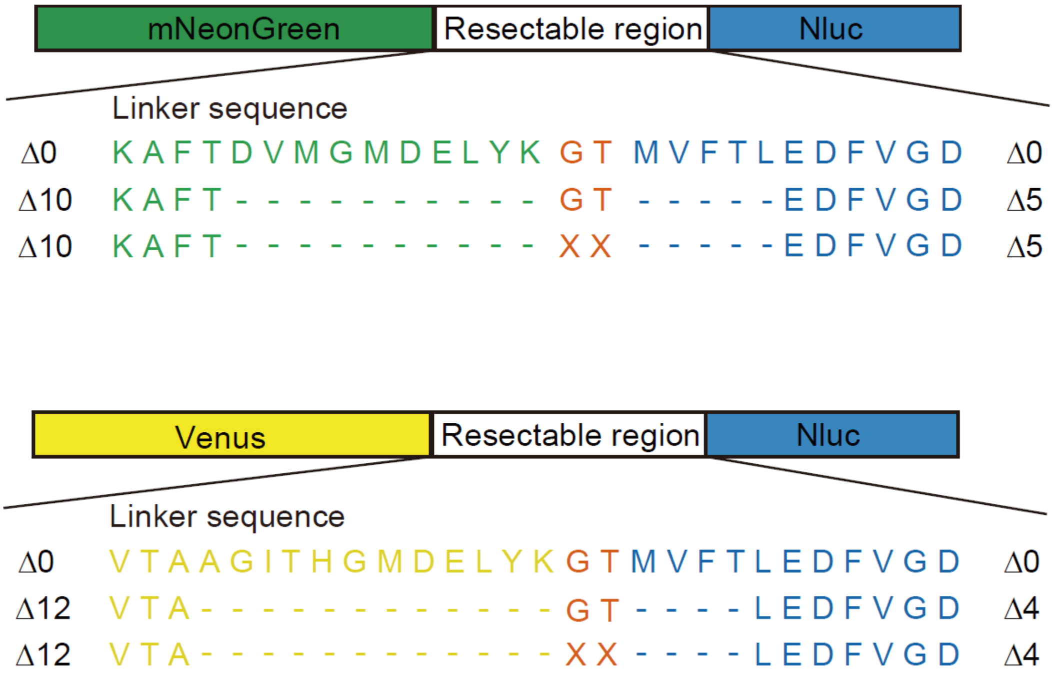

Using mNeonGreenΔC10-GT-NlucΔN5 and VenusΔC12-GT-NlucΔN4 as templates, we performed site-directed random mutagenesis at the linker region, -Gly-Thr-.

Supplementary Figure 4. Emission spectra of randomized linker variants in mNeonGreen-Nluc and Venus-Nluc fusion protein

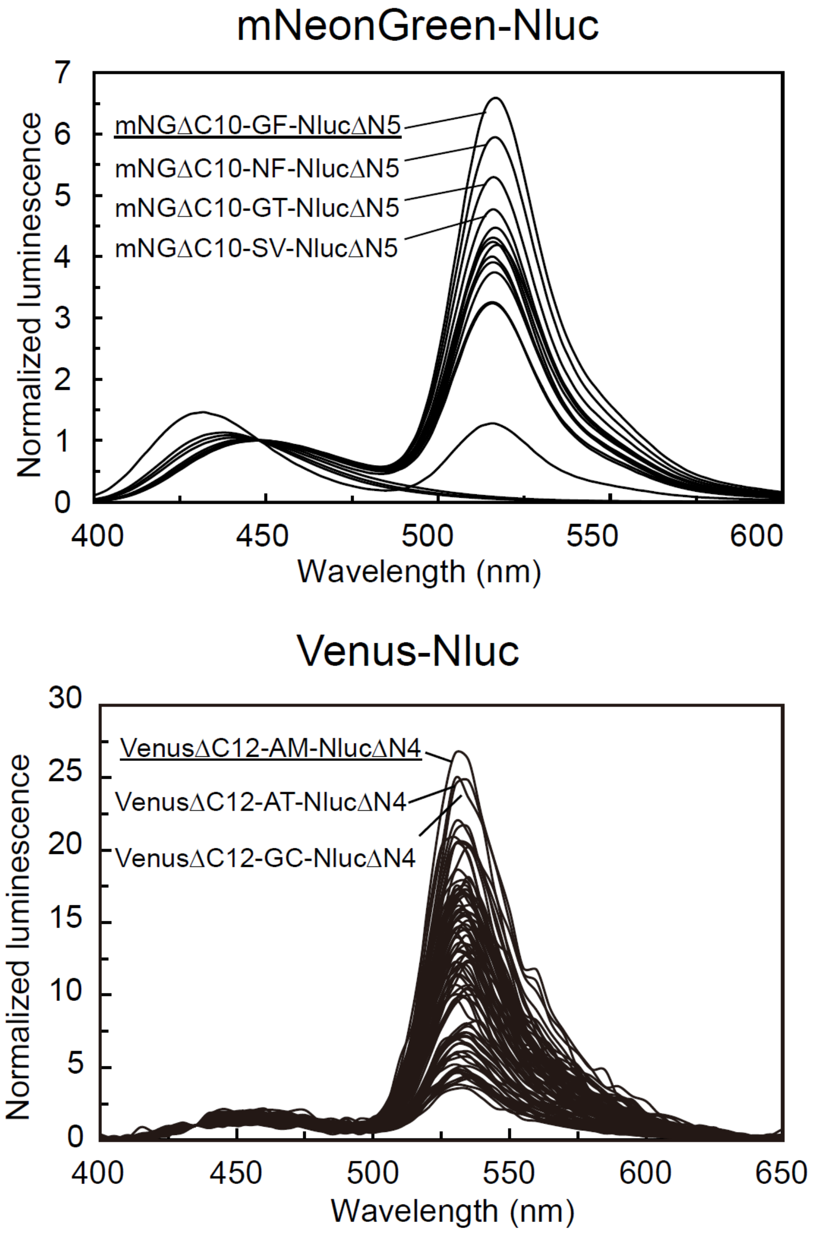

Luminescence spectra of *E. coli* suspension of colonies that showed bright luminescence signal on an agar plate at first screening. The spectra were normalized to the peak of Nluc emission intensity. Spectra were measured by micro-plate reader (SH-9000; Corona Electric). mNGΔC10-GF-NlucΔN5 (GeNL) and VenusΔC12-AM-NlucΔN4 (YeNL) exhibited the highest FRET efficiency.

Supplementary Figure 5. Design of linker library in mTurquoise2-Nluc and tdTomato-Nluc fusion protein

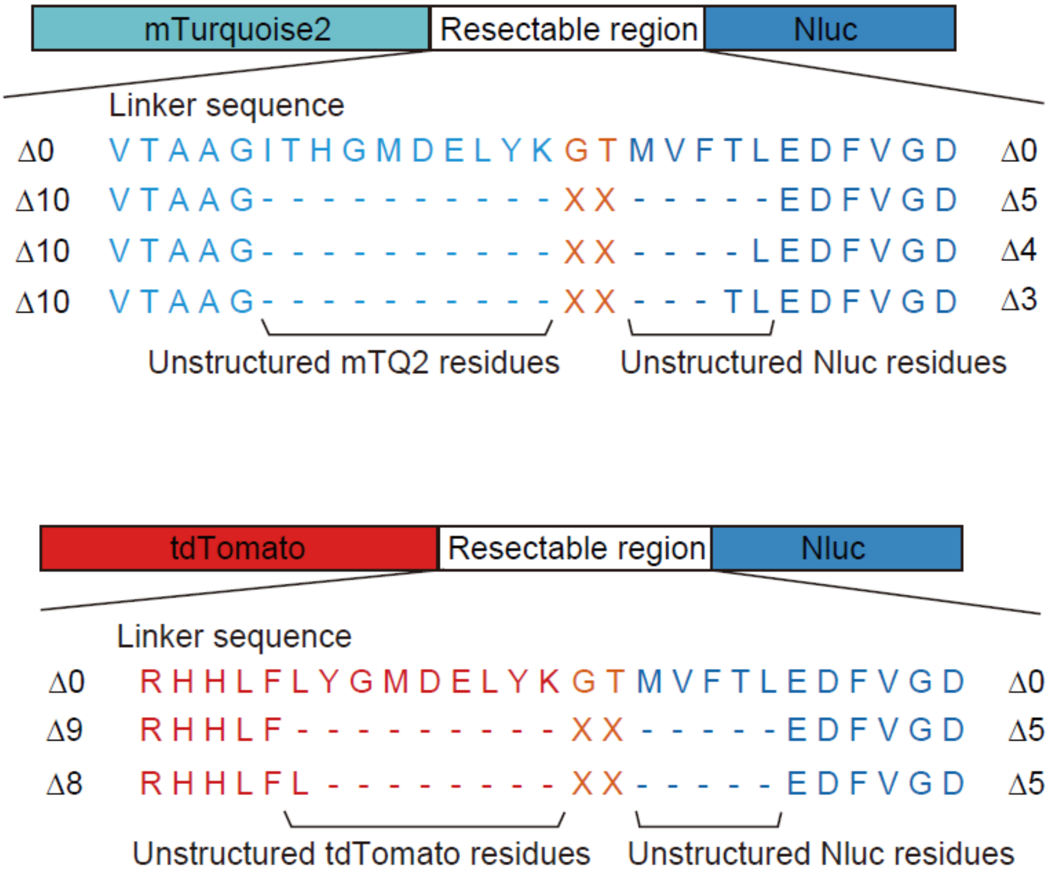

In the mTurquoise2-Nluc and tdTomato-Nluc pair, we created a series of linker libraries by systematically truncating resectable regions and randomizing two residues at the junction simultaneously. Those libraries with different linker lengths were mixed together and screened.

Supplementary Figure 6. Emission spectra of linker library in tdTomato-Nluc fusion protein

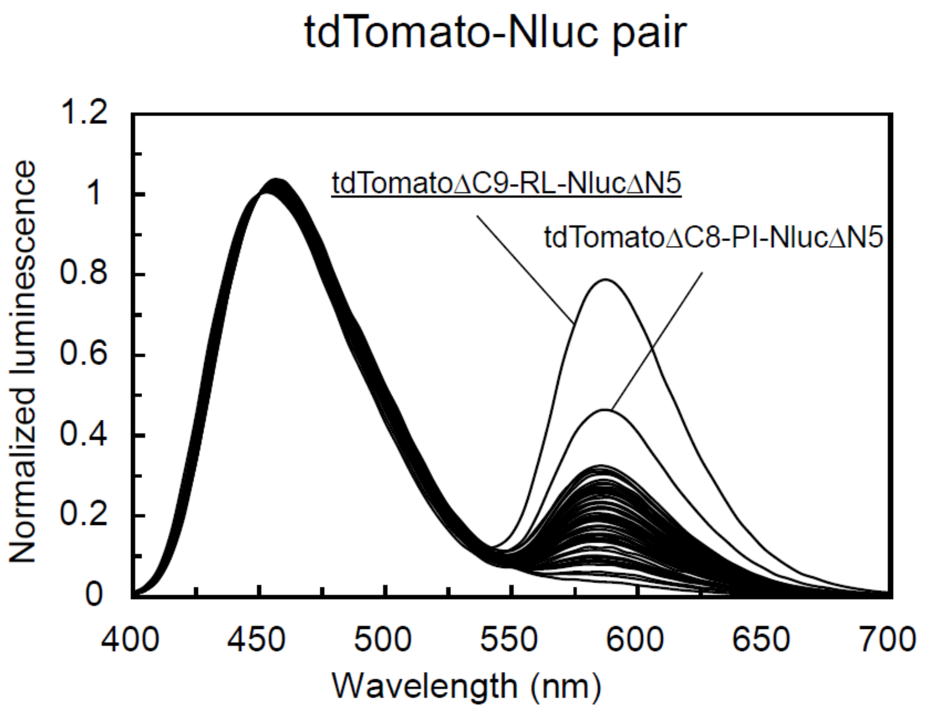

Luminescence spectra of *E. coli* suspension of colonies that showed bright luminescence signal on an agar plate at first screening. The spectra were normalized to the peak of Nluc emission intensity. Spectra were measured by micro-plate reader (SH-9000; Corona Electric). Among them, tdTomatoΔC9-RL-NlucΔN5 (ReNL) exhibited the highest FRET efficiency.

Supplementary Figure 7. Design of Orange eNano-lantern

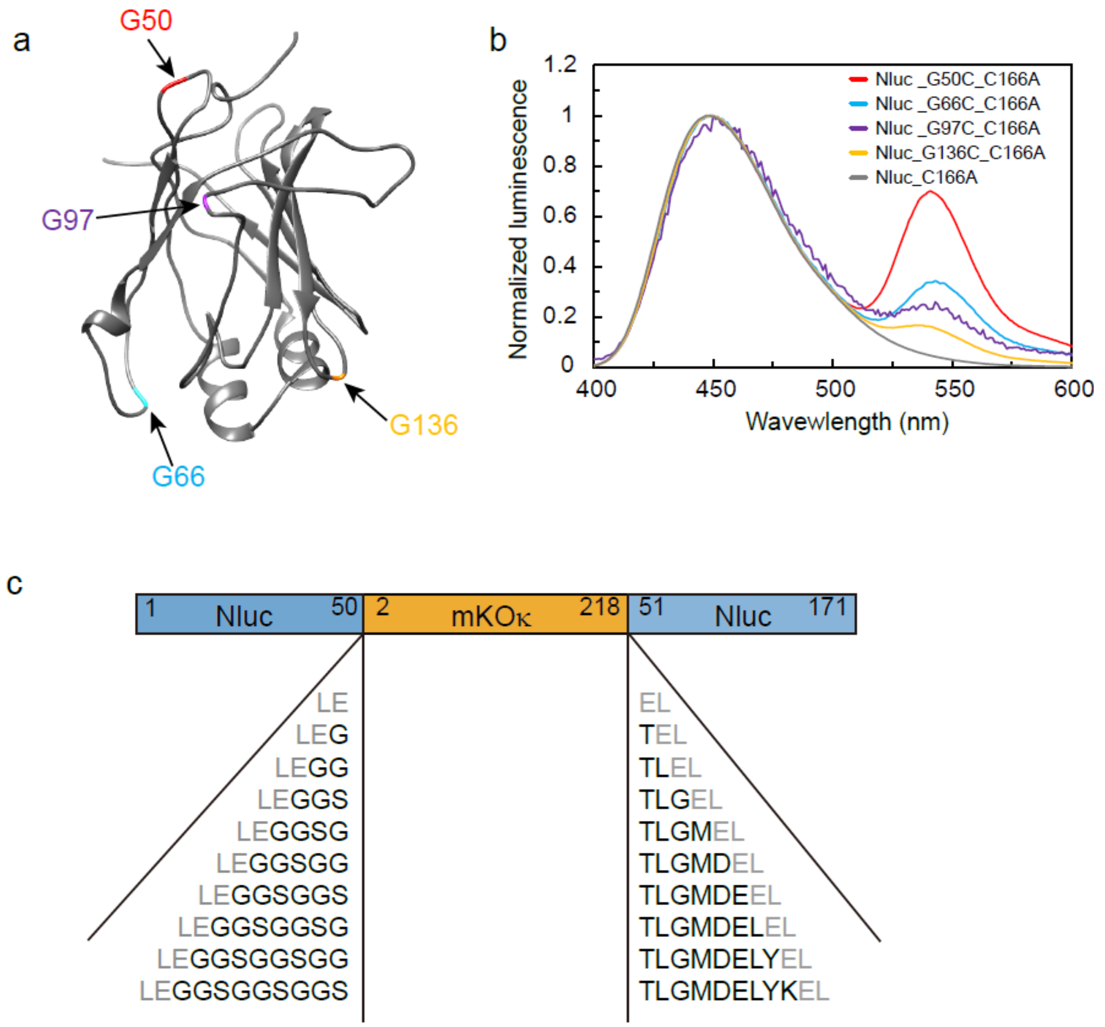

(a) 3D structure of Nluc predicted by I-TASSER with the positions of cysteine insertion (50, 66, 97 and 136th). (b) Luminescence spectra of Nluc conjugated with eosin-maleimide at designated cysteine. Each cysteine substitution was introduced to mutated Nluc whose intrinsic cysteine residue was substituted with alanine (C166A). (c) The flexible linker was introduced to flank mKOκ. Typical Glycine-rich linkers and mimics of GFP C-terminal 10 amino acids were employed as flexible linkers at the N-terminus and C-terminus of mKOκ, respectively. All possible combinations were screened.

88

Supplementary Figure 8. Emission spectra of FRET proteins based on the design of OeNL

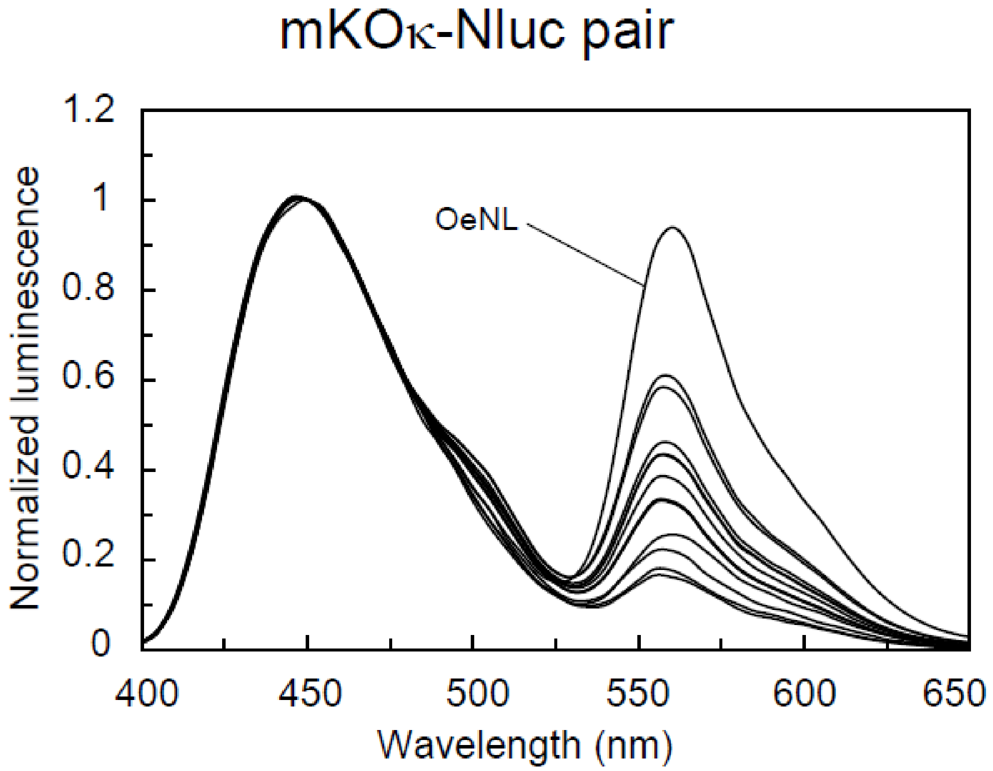

Luminescence spectra of *E. coli* colony suspensions showing the brightest luminescence signal on initial agar plate screening. The spectra measured by micro-plate reader (SH-9000; Corona Electric) and were normalized to the peak of Nluc emission intensity.

Supplementary Figure 9. Characterization of luminescent proteins

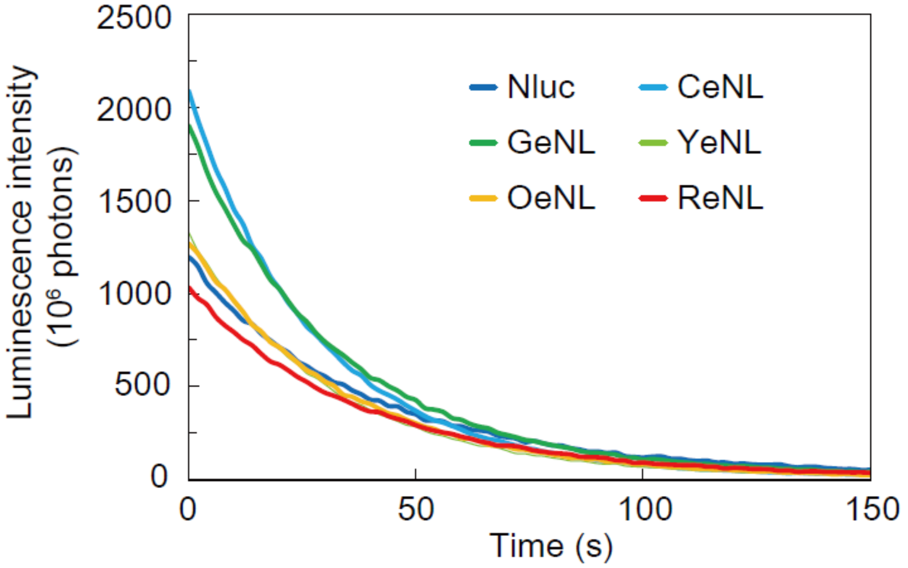

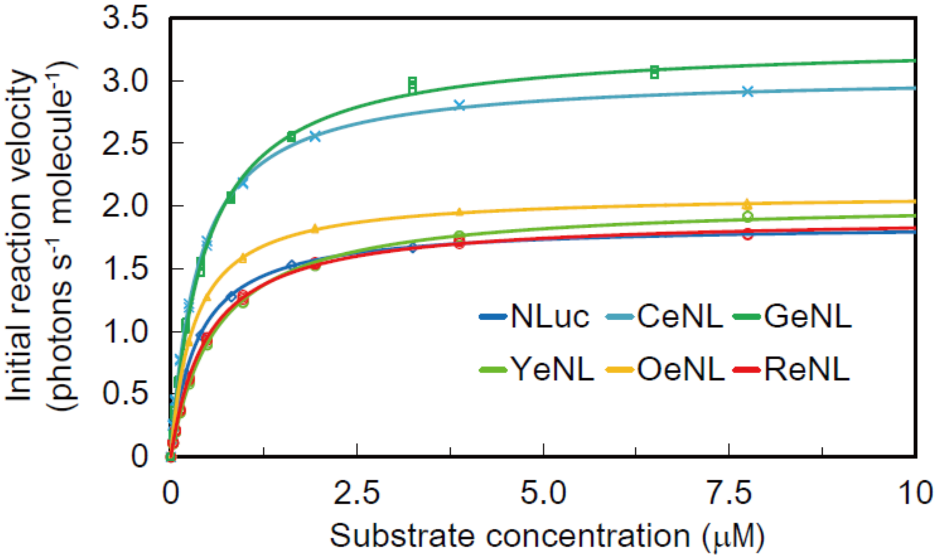

(a) Quantum yield was estimated from the integrated light output for 200 s until reaction of 500 pM of furimazine with 1 nM luminescent protein approached completion. Intensities were measured in triplicate, and data are presented as mean. (b) Kinetics parameters were estimated from the plot of the initial light output (first 12 second integration) versus the concentration of the furimazine. All intensities were measured in triplicate, and the average data were fitted to Michaelis-Menten equation. The results are summarized in Table S3.

Supplementary Figure 10. Single-cell imaging of HeLa cells expressing CeNL localized to various cellular compartments

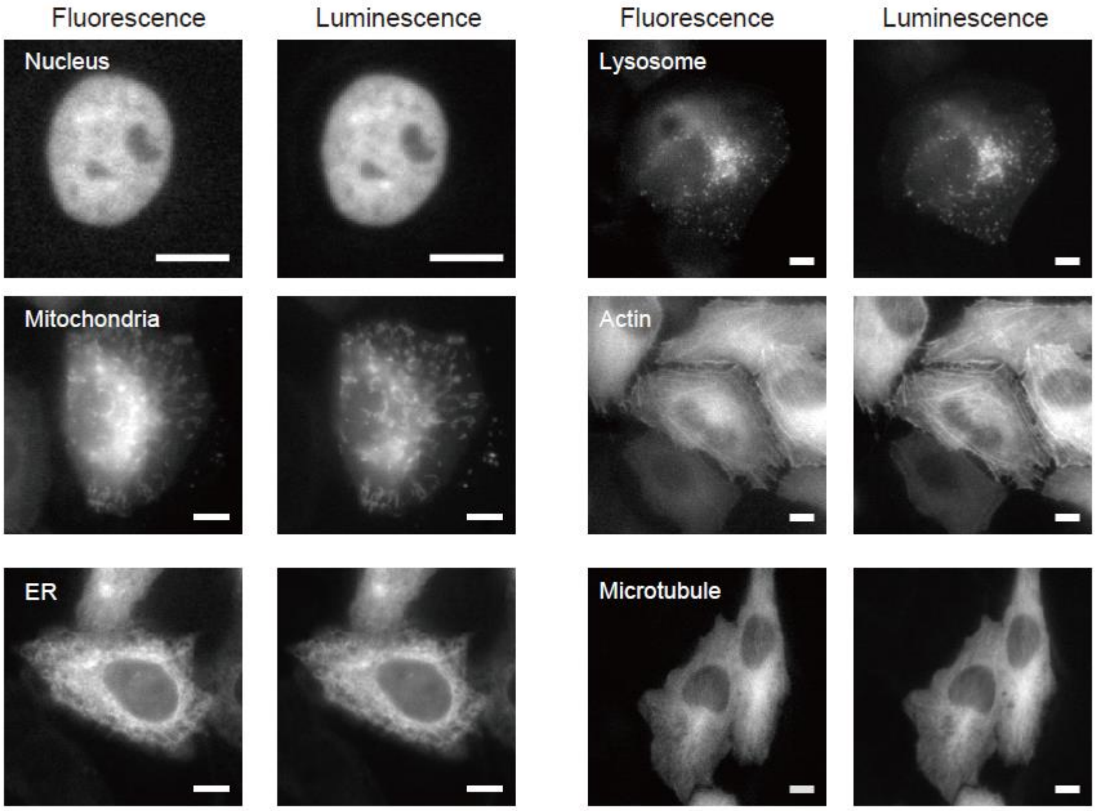

Fluorescence images (left) of mTurquoise2 moiety in CeNL and luminescence images (right) of CeNL localized to the nucleus, mitochondria, ER, lysosome, actin, and microtubule (exposure times for luminescence image acquisition times were 2 s, 20 s, 5 s, 10 s, 20 s, and 5 s, respectively). Scale bars, 10 μm.

Supplementary Figure 11. Single-cell imaging of HeLa cells expressing YeNL localized to various cellular compartments

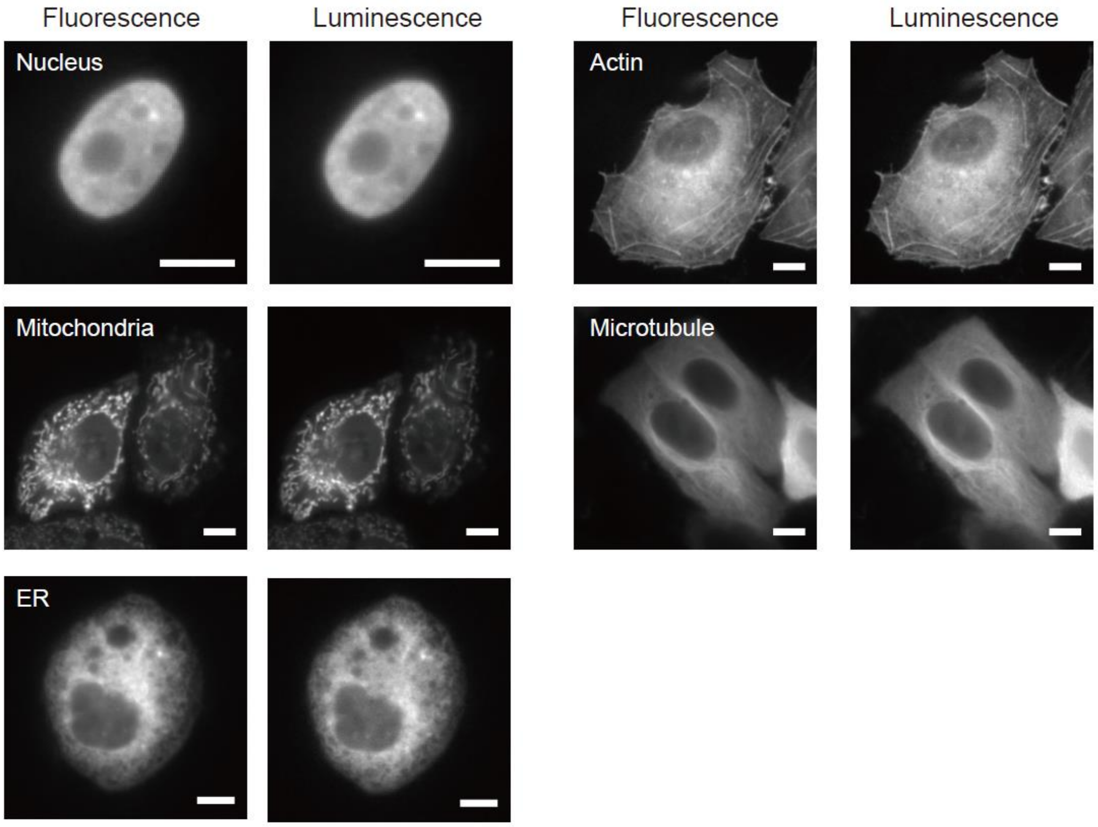

Fluorescence images (left) of the Venus moiety in YeNL and luminescence images (right) of YeNL localized to the nucleus, mitochondria, ER, actin, and microtubule (exposure times for luminescence images were 5 s, 5 s, 5 s, 5 s, and 10 s, respectively). Scale bars, 10 μm.

Supplementary Figure 12. Single-cell imaging of HeLa cells expressing OeNL localized to various cellular compartments

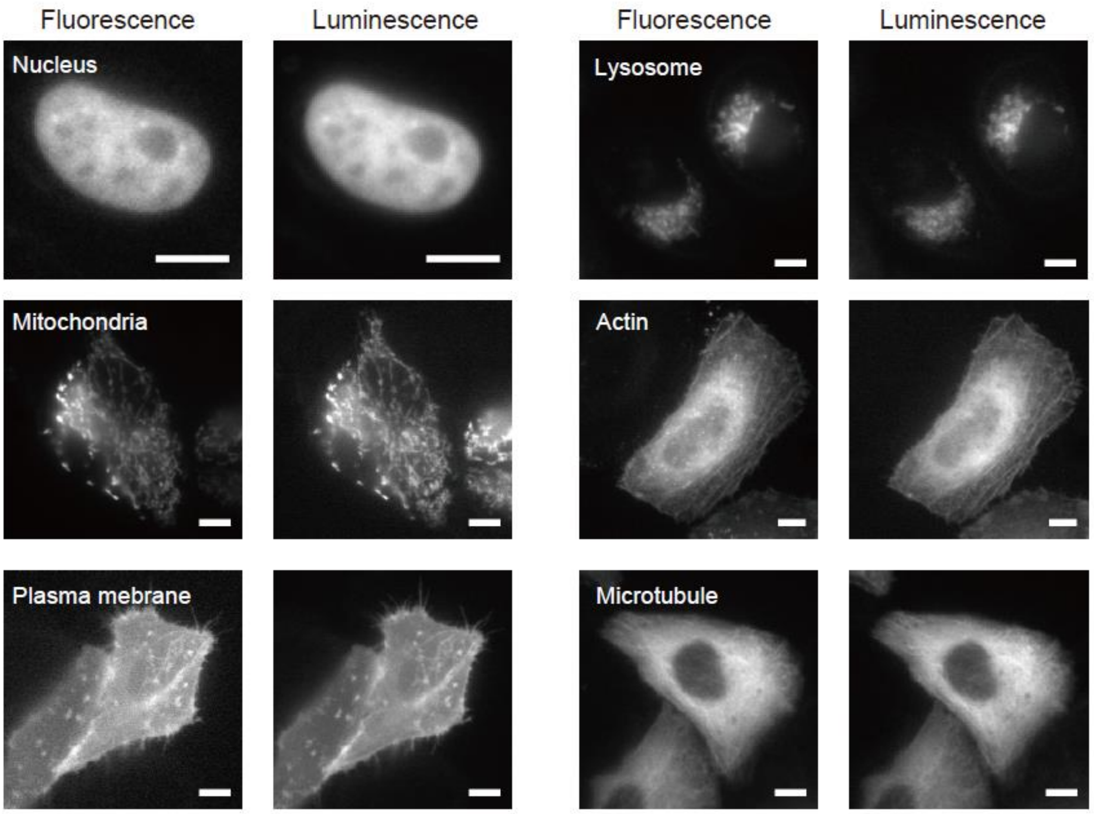

Fluorescence images (left) of mKOκ moiety in OeNL and luminescence images (right) of OeNL localized to the nucleus, mitochondria, plasma membrane, lysosome, actin, and microtubule (exposure time for luminescence images were 3 s, 10 s, 10 s, 5 s, 10 s, and 5 s, respectively). Scale bars, 10 μm.

Supplementary Figure 13. Single-cell imaging of HeLa cells expressing ReNL localized to various cellular compartments

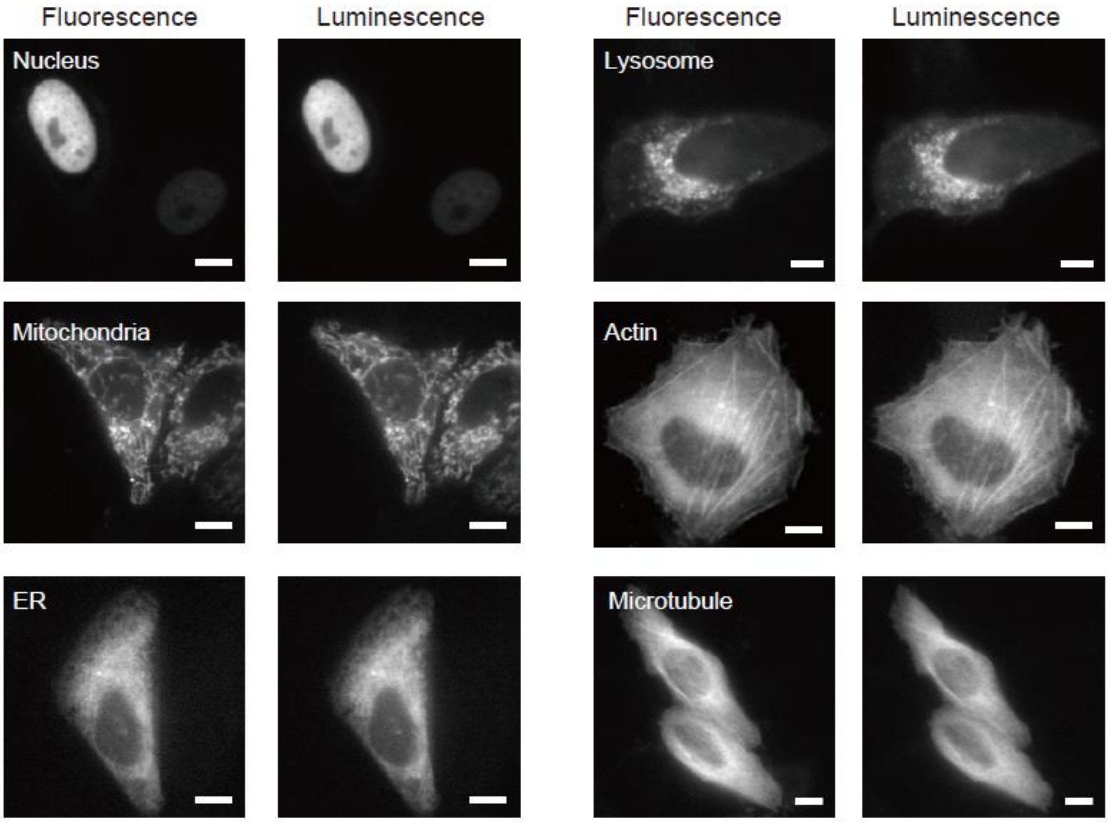

Fluorescence images (left) of the tdTomato moiety in ReNL and luminescence images (right) of ReNL localized to the nucleus, mitochondria, ER, lysosome, actin, and microtubule (exposure time for luminescence images were 3 s, 10 s, 10 s, 5 s, 10 s, and 5 s, respectively). Scale bars, 10 μm.

Supplementary Figure 14. Multi-color luminescence imaging with linear unmixing

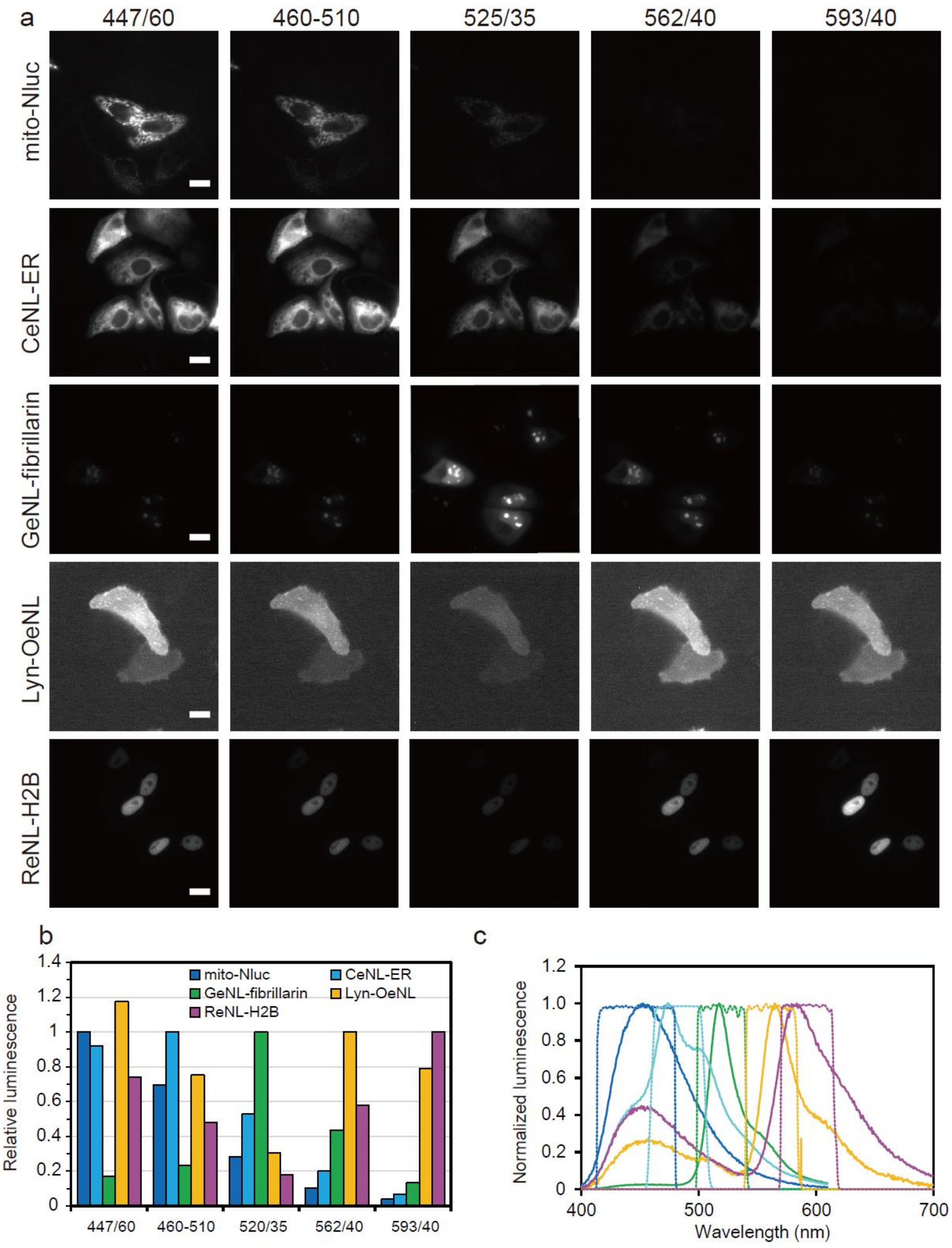

(a) HeLa cells expressing either mito-Nluc, CeNL-ER, GeNL-fibrillarin Lyn-OeNL or ReNL-H2B were imaged with five filters. Scale bars, 20 µm. (b) Linear spectral unmixing coefficient was determined from the control experiments as in **a**. (c) The emission spectra of Nluc, CeNL, GeNL, OeNL, and ReNL. Dashed line represents the emission filters used to acquire each luminescent protein signal.

Supplementary Figure 15. Identification of optimal insertion site of CaM-M13 into Nluc

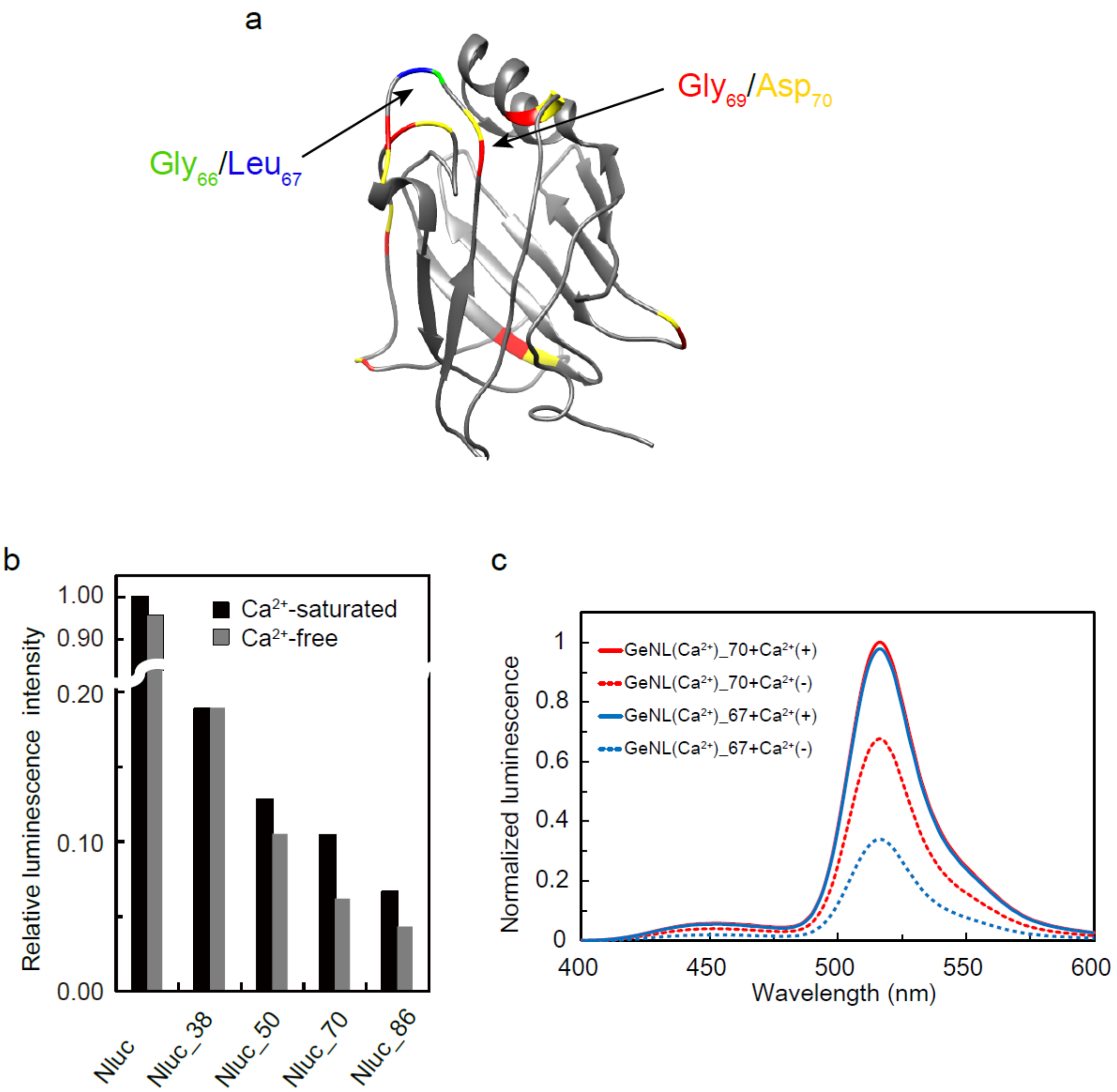

(a) 3D structure of Nluc predicted by I-TASSER with the candidate CaM-M13 insertion sites (37/38, 63/64, 69/70, 97/98, 103/104, 107/108, 121/122 and 148/149th with red and yellow color) and final optimal insertion site at 66/67th of GeNL(Ca^{2+}) with green and blue color. (b) Relative brightness of Nluc_CaM-M13 protein extracted from periplasmic region with or without Ca^{2+}. (c) Normalized luminescence spectra of GeNL(Ca^{2+})_67 and GeNL(Ca^{2+})_70 with or without Ca^{2+}.

Supplementary Figure 16. Direct comparison of GeNL(Ca^{2+}) and either GCaMP3 or Fura-2

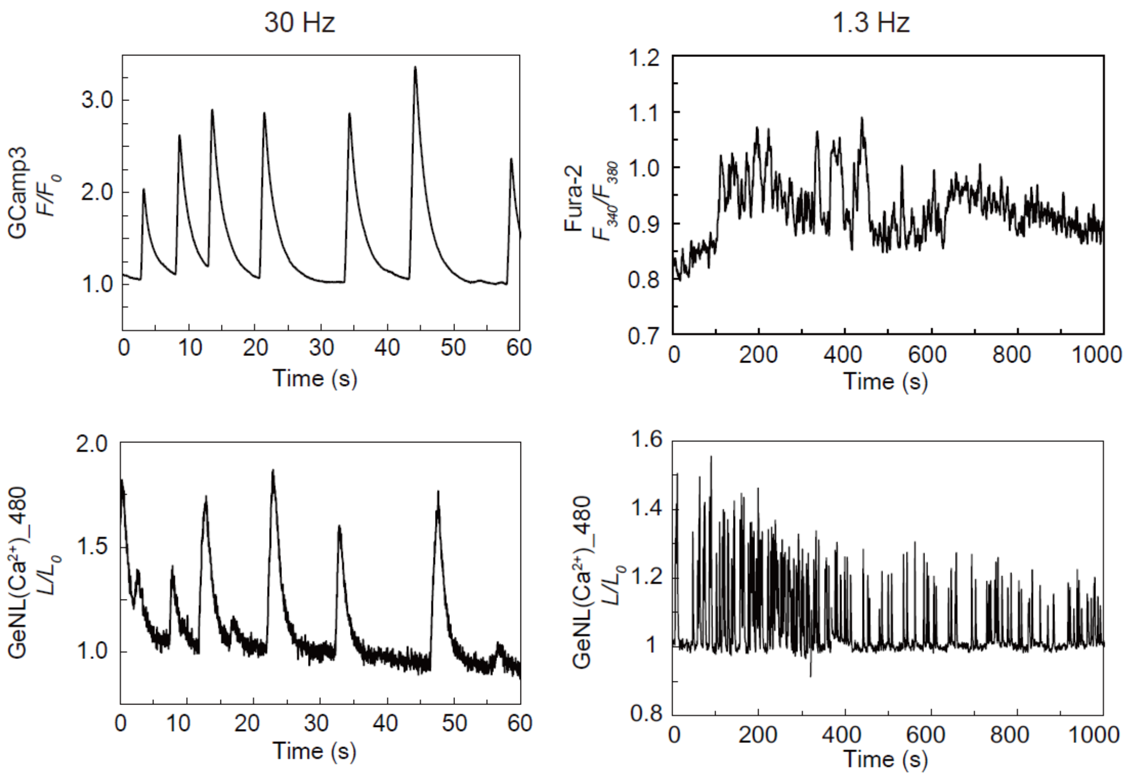

Spontaneous Ca^{2+} spiking in GH3 cells were monitored with the indicators (vertical axis) at designated frame rate (Upper). Illumination power was 130 mW cm^{-2} for GCaMP3, 100 mW cm^{-2} (384 nm) and 34 mW cm^{-2} (340 nm) for dual excitation ratio imaging of Fura-2. The background drift was manually subtracted using Origin7 software (OriginLab)

Supplementary Table 1. Enzymatic characteristics of luminescent proteins

	LQY (%)	K_m (μM)	V_{max} (photon s^{-1} $molecule^{-1}$)	k_{cat} (s^{-1})
Nluc	28 ± 3.0	0.36 ± 0.0038	1.9 ± 0.0061	6.6 ± 0.73
CeNL	42 ± 1.1	0.37 ± 0.0042	3.0 ± 0.010	7.1 ± 0.19
GeNL	45 ± 1.8	0.47 ± 0.010	3.3 ± 0.021	7.3 ± 0.30
YeNL	33 ± 1.1	0.61 ± 0.0088	2.0 ± 0.088	6.1 ± 0.34
OeNL	30 ± 0.48	0.31 ± 0.0025	2.1 ± 0.043	7.0 ± 0.18
ReNL	26 ± 1.0	0.49 ± 0.0064	1.9 ± 0.069	7.3 ± 0.20
Rluc8[a]	5.8 ± 0.63	2.0 ± 0.057	0.22 ± 0.0018	3.8 ± 0.12

LQY, luminescent quantum yield. Data are presented as mean ± s.d., n = 3.

[a] measured with 0.1% BSA

Supplementary Table 2. Affinity for Ca^{2+} of GeNL(Ca^{2+}) variants

	Linker of CaM and M13	Dynamic range / %	Relative luminescence	K_d / nM	Hill coefficient
GeNL(Ca^{2+})_520	104Q-2G	280	1	520	1.5
GeNL(Ca^{2+})_480	104Q-2GS	490	0.87	480	1.2
GeNL(Ca^{2+})_250	104Q-3GS	190	0.79	260	1.2
GeNL(Ca^{2+})_60	104E-4GS	270	0.76	56	1.1

Supplementary Table 3. Oligonucleotides used in this study

Name of primer	Oligonucleotide sequence (5' to 3')
F-BH1-G-gfp_1	TTGGATCCGATGGTGAGCAAGGGCGAGGAG
R-ER1-Nluc_171	ATGAATTCCGCCAGAATGCGTTCGCACAG
F-Kpn1-Nluc_2	GCCGGTACCGTCTTCACACTCGAAGATTTCG
F-Kpn1-Nluc_3	GCCGGTACCTTCACACTCGAAGATTTCGTTG
F-Kpn1-Nluc_4	GCCGGTACCACACTCGAAGATTTCGTTGGG
F-Kpn1-Nluc_5	GCCGGTACCCTCGAAGATTTCGTTGGGGAC
F-Kpn1-Nluc_6	GCCGGTACCGAAGATTTCGTTGGGGACTGGC
R-Kpn1-mNG_235	GCCGGTACCGTACAGCTCGTCCATGCCCATC
R-Kpn1-mNG_234	GCCGGTACCCAGCTCGTCCATGCCCATCAC
R-Kpn1-mNG_233	GCCGGTACCCTCGTCCATGCCCATCACATCG
R-Kpn1-mNG_232	GCCGGTACCGTCCATGCCCATCACATCGG
R-Kpn1-mNG_231	GCCGGTACCCATGCCCATCACATCGGTAAAG
R-Kpn1-mNG_230	GCCGGTACCGCCCATCACATCGGTAAAGGCC
R-Kpn1-mNG_229	GCCGGTACCCATCACATCGGTAAAGGCCTTTTGC
R-Kpn1-mNG_228	GCCGGTACCCACATCGGTAAAGGCCTTTTGC
R-Kpn1-mNG_227	GCCGGTACCATCGGTAAAGGCCTTTTGCCAC
R-Kpn1-mNG_226	GCCGGTACCGGTAAAGGCCTTTTGCCACTCC
F-XX-Nluc_6	NNKNNKGAAGATTTCGTTGGGGACTGG
R-mNG_226	GGTAAAGGCCTTTTGCCACTCC
R-Kpn1-gfp_229	ATGGTACCCCCGGCGGCGGTCACGAAC
F-XX-Nluc_5	NNKNNKGAAGATTTCGTTGGGGACTG
F-XX-Nluc_4	NNKNNKCTCGAAGATTTCGTTGGGG
F-XX-Nluc_3	NNKNNKACACTCGAAGATTTCGTTG
R-gfp_229	CCCGGCGGCGGTCACGAAC
F-Xhol-mKO_2	ATTCTCGAGGTGAGCGTGATCAA
F-Xhol-G-mKO_2	ATTCTCGAGGGCGTGAGCGTGATCAAGC
F-Xhol-GG-mKO_2	ATTCTCGAGGGCGGTGTGAGCGTGATCAAGC
F-Xhol-GGS-mKO_2	ATTCTCGAGGGCGGTAGCGTGAGCGTGATCAAGC
F-Xhol-GGSG-mKO_2	ATTCTCGAGGGCGGTAGCGGTGTGAGCGTGATCAAGC
F-Xhol-GGSGG-mKO-2	ATTCTCGAGGGCGGTAGCGGTGGCGTGAGCGTGATCAAGCCCGA
F-Xhol-GGSGGS-mKO-2	ATTCTCGAGGGCGGTAGCGGTGGCAGCGTGAGCGTGATCAAGCCCGA
F-Xhol-GGSGGSG-mKO-2	ATTCTCGAGGGCGGTAGCGGTGGCAGCGGAGTGAGCGTGATCAAGCCCGA
F-Xhol-GGSGGSGG-mKO-2	ATTCTCGAGGGCGGTAGCGGTGGCAGCGGAGGTGTGAGCGTGATCAAGCCCGA
F-Xhol-GGSGGSGGS-mKO-2	ATTCTCGAGGGCGGTAGCGGTGGCAGCGGAGGTAGCGTGAGCGTGATCAAGCCCGA
R-Sac1-mKO_218	ATTGAGCTCGGAGTGGGCCACGGCG
R-Sac1-T-mKO_218	ATTGAGCTCAGTGGAGTGGGCCACGGCG
R-Sac1-TL-mKO_218	ATTGAGCTCGAGAGTGGAGTGGGCCACGGCG
R-Sac1-TLG-mKO_218	ATTGAGCTCGCCGAGAGTGGAGTGGGCCACGGCG
R-Sac1-TLGM-mKO_218	ATTGAGCTCCATGCCGAGAGTGGAGTGGGCCACGGCG
R-Sac1-TLGMD-mKO_218	ATTGAGCTCGTCCATGCCGAGAGTGGAGTGGGCCACGGCG
R-Sac1-TLGMDE-mKO_218	ATTGAGCTCCTCGTCCATGCCGAGAGTGGAGTGGGCCACGGCG
R-Sac1-TLGMDEL-mKO_218	ATTGAGCTCCAGCTCGTCCATGCCGAGAGTGGAGTGGGCCACGGCG
R-Sac1-TLGMDELY-mKO_218	ATTGAGCTCGTACAGCTCGTCCATGCCGAGAGTGGAGTGGGCCACGGCG
R-Sac1-TLGMDELYK-mKO_218	ATTGAGCTCCTTGTACAGCTCGTCCATGCCGAGAGTGGAGTGGGCCACGGCG
R-Kpn1-tdTA_467	GCCGGTACCCAGGAACAGGTGGTGGCGGCC
F-Nluc	GGGACTGGCGACAGACAGCCG
R-Nluc_6-XX-tdTA_467	CAACGAAATCTTCMNNMNNCAGGAACAGGTGGTG
R-Nluc_6-XX-tdTA_466	CAACGAAATCTTCMNNMNNGAACAGGTGGTG
R-Nluc_6-XX-tdTA_465	CAACGAAATCTTCMNNMNNCAGGTGGTGGCG
F-Sac1-Nluc_38	GCCGAGCTCGTGTCCGTAACTCCGATCCAAAG
R-Nco1-Nluc_37	GAACCATGGCCCGAGATTCTGAAACAAACTG
F-Sac1-Nluc_64	GCCGAGCTCTATGAAGGTCTGAGCGGCGAC
R-Nco1-Nluc_63	GAACCATGGCGGGATGATGACATGGATGTC
F-Sac1-Nluc_67	GCCGAGCTCCTGAGCGGCGACCAAATGGGC
R-Nco1-Nluc_66	GAACCATGGACCTTCATACGGATGATGAC

F-Sac1-Nluc_70	GCCGAGCTCGACCAAATGGGCCAGATC
R-Nco1-Nluc_69	GAACCATGGCCGCTCAGACCTTCATACGG
F-Sac1-Nluc_98	GCCGAGCTCACACTGGTAATCGACGGGG
R-Nco1-Nluc_97	GAACCATGGCCATAGTGCAGGATCACC
F-Sac1-Nluc_104	GCCGAGCTCGTTACGCCGAACATGATC
R-Nco1-Nluc_103	GAACCATGGCCCGTCGATTACCAGTGTG
F-Sac1-Nluc_108	GCCGAGCTCATGATCGACTATTTCGGACGG
R-Nco1-Nluc_107	GAACCATGGGTTCGGCGTAACCCCGTC
F-Sac1-Nluc_122	GCCGAGCTCTTCGACGGCAAAAAGATCACTG
R-Nco1-Nluc_121	GAACCATGGCACGGCGATGCCTTCATAC
F-Sac1-Nluc_149	GCCGAGCTCGGCTCCCTGCTGTTCCGAG
R-Nco1-Nluc_148	GAACCATGGGTCGGGGTTGATCAGGCGCTC
F-BH1-koz-hmNG	ATGGATCCCGCCACCATGGTGTCCAAGGGCGAAGAG
F-BH1-G-hmNG	ATGGATCCGATGGTGTCCAAGGGCGAAG
F-BH1-hmNG	ATGGATCCATGGTGTCCAAGGGCGAAGAG
F-Kpn1-hmNG_2	ATGGTACCGTGTCCAAGGGCGAAGAG
F-Hind3-koz-hmNG_1	ATAAGCTTCGCCACCATGGTGTCCAAGGGCGAAGAG
F-Sal1-link-hmNG_1	ATGTCGACGGTACCGCGGGCCCGGGATCCAATGGTGTCCAAGGGCGAAGAG
F-Nhe1-koz-hmNG_1	ATGCTAGCCGCCACCATGGTGTCCAAGGGCGAAGAGG
R-Xho1-x-Nluc_171	ATCTCGAGTTACGCCAGAATGCGTTCGCACAG
R-ER1-x-Nluc_171	ATGAATTCTTACGCCAGAATGCGTTCGCACAG
R-Nluc_171-ER1	ATGAATTCCGCCAGAATGCGTTCGCACAG
R-Kpn1-Nluc_171	TATGGTACCCGCCAGAATGCGTTCGCACAG
R-Kpn1-GGSG-Nluc_171	ATGGTACCGCCTGATCCACCCGCCAGAATGCGTTCGCACAG
R-Not1-x-NLuc_171	ATGCGGCCGCTTACGCCAGAATGCGTTCGC
R-Bgl2-Nluc_171	ATTAGATCTCGCCAGAATGCGTTCGCACAG
F-Sal1-GS10 linker	TCGACCGGATCTGGCGGCGGAGGAAGCGGAGGG
R-Sal1-GS10 linker	TCGACCCTCCGCTTCCTCCGCCGCCAGATCCGG
F- BH1-koz-gfp_1	ATGGATCCCCACCATGGTGAGCAAGGGCGAGGAG
F-Hind3-koz-gfp_1	ATAAGCTTCGCCACCATGGTGAGCAAGGGCGAGGAG
F- BH1-gfp_1	TTGGATCCATGGTGAGCAAGGGCGAGGAG
F-GS-VCL_2	GGAGGCGGAGGATCAGGCGGATCTGGGCCCGTCTTCCACACGCGCAC
F-Kpn1-GS	ATGGTACCGGCGGCGGAGGAAGCGGAGGCGGAGGATCAGGCGGATC
R-ER1-x-VCL_1066	ATGAATTCTTACTGATACCATGGGGTCTTTC
F-Hind3-koz-LAMP_1	ATAAGCTTCGCCACCATGGCGGCCCCGGGCGCCCGG
R-Kpn1-LAMP_407	ATGGTACCGATGGTCTGATAGCCCGCGTGAC
F-BH1-Nluc_1	A GGATCC G ATGGTCTTCACACTCGAAGATTTCGTTGGGGACTGG
F-BH1-NLuc_1	ATGGATCCATGGTCTTCACACTCGAAG
F-Hind3-koz-Nluc_1	ATAAGCTTCGCCACCATGGTCTTCACACTCGAAG
F-ER1-Nluc_1	AGAATTCATGGTCTTCACACTCGAAGATTTCGTTGGGGACTGG
Nluc_C166A	GGCTGGCGGCTGGCCGAACGCATTCTG
F-Nluc_G50C	TGCGAAAATGGGCTGAAGATC
R-Nluc_49	GCTCAGGACAATCCTTTG
F-Nluc_G66C	TGCCTGAGCGGCGACCAAATG
R-Nluc_65	TTC ATACGGGATGATGAC
F-Nluc_G97C	TGCACACTGGTAATCGACGGG
R-Nluc_96	ATAGTGCAGGATCACC
F-Nluc_G136C	TGCAACAAAATTATCGACGAGC
R-Nluc_135	GTTCCACAGGGTCCCTG

Supplementary Note 1

Consideration of the insertion site of mKOκ

To achieve efficient FRET from Nluc to mKOκ, we decided to insert mKOκ into the loop region of Nluc. To find the closest position of the luciferin-binding site, we substituted a glycine residue with a cysteine residue at four putative flexible loop regions of Nluc (50th, 66th, 97th, and 136th) and conjugated eosin, a yellowish orange fluorescent dye, with each cysteine residue via the maleimide functional group (**Supplementary Fig. 7a and 7b**). The Nluc conjugated with eosin at the 50th residue exhibited the highest FRET efficiency among tested constructs, suggesting that the luciferin binding site is close to the 50th residue of Nluc. Thus, we inserted mKOκ in between Gly_{50} and Glu_{51} of Nluc and generated a small library with flexible linker amino acids inserted at the N- and C-termini of the mKOκ domain (**Supplementary Fig. 7c**).

Supplementary Note 2

Estimation of photon number that single GeNL molecule emits

Based on enzymatic parameters, the photon number that can be emitted by a single molecule of GeNL during camera exposure was 590 ± 3.8 photons (3.3 photons s^{-1} x 180 s, mean ± SD, n = 3). The reported photon detection efficiency of an objective lens (NA 1.45, x100 magnification, oil immersion), similar to that used is 15% when the specimen is located at a height of 200 nm from the glass-water interface[1] (typical thickness of agarose used here[2]). Thus the number of photons reaching the camera is anticipated to be 89 ± 0.57 photons (mean ± SD). Separately, we calculated the total number of counts collected from single luminescence spots and then converted this number to the number of photons using 5.8 conversion efficiency, 1200 electron multiplication, an ADC gain setting of 5, and a quantum efficiency of 0.9 at 520 nm (ImagEM, Hamamatsu Photonics). The number of detected photons was 75 ± 30 photons (mean ± SD, n = 919).

Supplementary Note3

Consideration of the insertion site for eNL-based indicators

To develop a Ca^{2+} indicator based on GeNL, we adopted the intramolecular complementation of split luciferase, in which the sensor domain of a bioactive molecule is inserted into luciferase. The conformational change of the sensor domain by analyte binding induces the reconstitution of the split luciferase domains. We chose a fusion protein of calmodulin and M13 as a Ca^{2+} sensing domain. To create high-performance CSL-based indicators, it is important to design an appropriate insertion site that allows the split Luciferase to display a large dynamic range in signal change, an intensity bright enough for imaging. Thus we systematically screened and identified the appropriate sites of Nluc for CaM-M13 insertion. Through the use of transposon-based mutagenesis[3], we constructed a library of Nluc gene variants that contained 15 base pairs of DNA inserted at a random location, followed by expression in *E. coli*[3]. We screened several thousand individual clones and picked 40 based on the luminescence signal intensity. Subsequent DNA sequencing revealed the insertion sites within the Nluc (37/38, 63/64, 69/70, 97/98, 103/104, 107/108, 121/122 and 148/149th residue) at which five amino acids were inserted without obliterating the intrinsic activity of Nluc (Supplementary Fig. 16a**)**. Next we inserted the CaM-M13 domain into the identified sites within Nluc. As a result, CaM-M13 insertion in between Gly_{69} and Asp_{70} of Nluc yielded a 60% signal increase upon Ca^{2+} binding (Supplementary Fig. 16b). The construct, in which CaM-M13 was inserted in between Gly_{69} and Asp_{70} of the Nluc moiety in GeNL, showed almost the same signal change (60%). The spectrum profile was unchanged upon Ca^{2+} binding, indicating that the eNL-based Ca^{2+} indicator indeed used a Ca^{2+}-dependent CSL mechanism whose emission color was changed by FRET, but which was insensitive to Ca^{2+}. We searched for a more optimal insertion sites around Gly_{69}/Asp_{70}, and identified that the insertion in between Gly_{66} and Leu_{67} of the Nluc moiety in GeNL yielded a 180% signal increase upon Ca^{2+} binding (Supplementary Fig. 16c). Thus we named this construct GeNL(Ca^{2+}).

Supplementary Note4

Nucleotide sequences of eNL constructs used in this study are listed below. Acceptor fluorescent proteins is highlighted in yellow, Nluc highlighted in cyan, CaM-M13 highlighted in green.

>CeNL

ATGGTGAGCAAGGGCGAGGAGCTGTTCACCGGGGTGGTGCCCATCCTGGTC
GAGCTGGACGGCGACGTAAACGGCCACAAGTTCAGCGTGTCCGGCGAGGGC
GAGGGCGATGCCACCTACGGCAAGCTGACCCTGAAGTTCATCTGCACCACCG
GCAAGCTGCCCGTGCCCTGGCCCACCCTCGTGACCACCCTGTCCTGGGGCGT
GCAGTGCTTCGCCCGCTACCCCGACCACATGAAGCAGCACGACTTCTTCAAG
TCCGCCATGCCCGAAGGCTACGTCCAGGAGCGCACCATCTTCTTCAAGGACG
ACGGCAACTACAAGACCCGCGCCGAGGTGAAGTTCGAGGGCGACACCCTGG
TGAACCGCATCGAGCTGAAGGGCATCGACTTCAAGGAGGACGGCAACATCC
TGGGGCACAAGCTGGAGTACAACTACTTTAGCGACAACGTCTATATCACCGC
CGACAAGCAGAAGAACGGCATCAAGGCCAACTTCAAGATCCGCCACAACAT
CGAGGACGGCGGCGTGCAGCTCGCCGACCACTACCAGCAGAACACCCCCAT
CGGCGACGGCCCCGTGCTGCTGCCCGACAACCACTACCTGAGCACCCAGTC
CAAGCTGAGCAAAGACCCCAACGAGAAGCGCGATCACATGGTCCTGCTGGA
GTTCGTGACCGCCGCCGGGTTGCATACACTCGAAGATTTCGTTGGGGACTGG
CGACAGACAGCCGGCTACAACCTGGACCAAGTCCTTGAACAGGGAGGTGTG
TCCAGTTTGTTTCAGAATCTCGGGGTGTCCGTAACTCCGATCCAAAGGATTGT
CCTGAGCGGTGAAAATGGGCTGAAGATCGACATCCATGTCATCATCCCGTATG
AAGGTCTGAGCGGCGACCAAATGGGCCAGATCGAAAAAATTTTTAAGGTGG
TGTACCCTGTGGATGATCATCACTTTAAGGTGATCCTGCACTATGGCACACTG
GTAATCGACGGGGTTACGCCGAACATGATCGACTATTTCGGACGGCCGTATGA
AGGCATCGCCGTGTTCGACGGCAAAAAGATCACTGTAACAGGGACCCTGTG
GAACGGCAACAAAATTATCGACGAGCGCCTGATCAACCCCGACGGCTCCCTG
CTGTTCCGAGTAACCATCAACGGAGTGACCGGCTGGCGGCTGTGCGAACGCA
TTCTGGCGTAA

>GeNL

ATGGTGTCCAAGGGCGAAGAGGACAACATGGCCAGCCTGCCTGCCACCCAC
GAGCTGCACATCTTCGGCAGCATCAACGGCGTGGACTTCGACATGGTGGGAC
AGGGCACCGGCAACCCCAACGACGGCTACGAGGAACTGAACCTGAAGTCCA
CAAAGGGCGACCTGCAGTTCAGCCCCTGGATTCTGGTGCCCCACATCGGCTA
CGGCTTCCACCAGTACCTGCCCTACCCCGACGGCATGAGCCCTTTCCAGGCC
GCTATGGTGGATGGCAGCGGCTACCAGGTGCACCGGACCATGCAGTTTGAGG
ACGGCGCCAGCCTGACCGTGAACTACCGGTACACATACGAGGGCAGCCACAT
CAAGGGCGAGGCCCAAGTGAAGGGCACAGGCTTTCCAGCCGACGGCCCCGT
GATGACCAATAGCCTGACAGCCGCCGACTGGTGCAGAAGCAAGAAAACCTA
CCCCAATGACAAGACCATCATCAGCACCTTCAAGTGGTCCTACACCACCGGC
AATGGCAAGCGGTACAGAAGCACCGCCCGGACCACCTACACCTTCGCCAAA
CCTATGGCCGCCAACTACCTGAAGAACCAGCCTATGTACGTGTTCCGCAAGA
CCGAGCTGAAGCACTCCAAGACAGAACTGAACTTCAAAGAGTGGCAGAAA
GCCTTCACCGGGTTTGAAGATTTCGTTGGGGACTGGCGACAGACAGCCGGCT
ACAACCTGGACCAAGTCCTTGAACAGGGAGGTGTGTCCAGTTTGTTTCAGAA
TCTCGGGGTGTCCGTAACTCCGATCCAAAGGATTGTCCTGAGCGGTGAAAAT
GGGCTGAAGATCGACATCCATGTCATCATCCCGTATGAAGGTCTGAGCGGCG

ACCAAATGGGCCAGATCGAAAAAATTTTTAAGGTGGTGTACCCTGTGGATGA
TCATCACTTTAAGGTGATCCTGCACTATGGCACACTGGTAATCGACGGGGTTA
CGCCGAACATGATCGACTATTTCGGACGGCCGTATGAAGGCATCGCCGTGTTC
GACGGCAAAAAGATCACTGTAACAGGGACCCTGTGGAACGGCAACAAAATT
ATCGACGAGCGCCTGATCAACCCCGACGGCTCCCTGCTGTTCCGAGTAACCA
TCAACGGAGTGACCGGCTGGCGGCTGTGCGAACGCATTCTGGCGTAA

>YeNL
ATGGTGAGCAAGGGCGAGGAGCTGTTCACCGGGGTGGTGCCCATCCTGGTC
GAGCTGGACGGCGACGTAAACGGCCACAAGTTCAGCGTGTCCGGCGAGGGC
GAGGGCGATGCCACCTACGGCAAGCTGACCCTGAAGCTGATCTGCACCACC
GGCAAGCTGCCCGTGCCCTGGCCCACCCTCGTGACCACCCTGGGCTACGGCC
TGCAGTGCTTCGCCCGCTACCCCGACCACATGAAGCAGCACGACTTCTTCAA
GTCCGCCATGCCCGAAGGCTACGTCCAGGAGCGCACCATCTTCTTCAAGGAC
GACGGCAACTACAAGACCCGCGCCGAGGTGAAGTTCGAGGGCGACACCCTG
GTGAACCGCATCGAGCTGAAGGGCATCGACTTCAAGGAGGACGGCAACATC
CTGGGGCACAAGCTGGAGTACAACTACAACAGCCACAACGTCTATATCACCG
CCGACAAGCAGAAGAACGGCATCAAGGCCAACTTCAAGATCCGCCACAACA
TCGAGGACGGCGGCGTGCAGCTCGCCGACCACTACCAGCAGAACACCCCCA
TCGGCGACGGCCCCGTGCTGCTGCCCGACAACCACTACCTGAGCTACCAGTC
CGCCCTGAGCAAAGACCCCAACGAGAAGCGCGATCACATGGTCCTGCTGGA
GTTCGTGACCGCCGCGATGCTCGAAGATTTCGTTGGGGACTGGCGACAGACA
GCCGGCTACAACCTGGACCAAGTCCTTGAACAGGGAGGTGTGTCCAGTTTGT
TTCAGAATCTCGGGGTGTCCGTAACTCCGATCCAAAGGATTGTCCTGAGCGG
TGAAAATGGGCTGAAGATCGACATCCATGTCATCATCCCGTATGAAGGTCTGA
GCGGCGACCAAATGGGCCAGATCGAAAAAATTTTTAAGGTGGTGTACCCTGT
GGATGATCATCACTTTAAGGTGATCCTGCACTATGGCACACTGGTAATCGACG
GGGTTACGCCGAACATGATCGACTATTTCGGACGGCCGTATGAAGGCATCGC
CGTGTTCGACGGCAAAAAGATCACTGTAACAGGGACCCTGTGGAACGGCAA
CAAAATTATCGACGAGCGCCTGATCAACCCCGACGGCTCCCTGCTGTTCCGA
GTAACCATCAACGGAGTGACCGGCTGGCGGCTGTGCGAACGCATTCTGGCGT
AA

>OeNL
ATGGTCTTCACACTCGAAGATTTCGTTGGGGACTGGCGACAGACAGCCGGCT
ACAACCTGGACCAAGTCCTTGAACAGGGAGGTGTGTCCAGTTTGTTTCAGAA
TCTCGGGGTGTCCGTAACTCCGATCCAAAGGATTGTCCTGAGCGGTCTCGAG
GGCGGTAGCGGTGGCAGCGTGAGCGTGATCAAGCCCGAGATGAAGAAGGTG
GAGGACGCCGTGGCCCACTCCACTCTCGAGGGCGTGAGCGTGATCAAGCCC
GAGATGAAGATGAGGTACTACATGGACGGCTCCGTCAATGGGCATGAGTTCA
CAATCGAGGGTGAGGGCACAGGCAGACCTTACGAGGGACATCAGGAGATGA
CACTGCGCGTCACAATGGCCGAGGGCGGGCCAATGCCTTTCGCCTTCGACCT
GGTGTCCCACGTGTTCTGTTACGGCCACAGAGTGTTTACTAAATATCCAGAAG
AGATCCCAGACTATTTCAAGCAGGCCTTTCCTGAGGGCCTGTCCTGGGAGAG
GTCCCTGGAGTTCGAGGACGGCGGCTCCGCCTCCGTGAGCGCCCACATCAG
CCTGAGGGGCAACACCTTCTACCACAAGTCCAAGTTCACCGGCGTGAACTTC
CCCGCCGACGGCCCCATCATGCAGAACCAGAGCGTGGACTGGGAGCCCTCC
ACCGAGAAGATCACCGCCAGCGACGGCGTGCTGAAGGGCGACGTGACCATG
TACCTGAAGCTGGAGGGCGGCGGCAACCACAAGTGCCAGATGAAGACCACC
TACAAGGCCGCCAAGGAGATCCTGGAGATGCCCGGCGACCACTACATCGGCC
ACAGGCTGGTGAGGAAGACCGAGGGCAACATCACCGAGCAGGTGGAGGAC
GCCGTGGCCCACTCCGAGCTCGAAAATGGGCTGAAGATCGACATCCATGTCA
TCATCCCGTATGAAGGTCTGAGCGGCGACCAAATGGGCCAGATCGAAAAAAT
TTTTAAGGTGGTGTACCCTGTGGATGATCATCACTTTAAGGTGATCCTGCACT

ATGGCACACTGGTAATCGACGGGGTTACGCCGAACATGATCGACTATTTCGG
ACGGCCGTATGAAGGCATCGCCGTGTTCGACGGCAAAAAGATCACTGTAACA
GGGACCCTGTGGAACGGCAACAAAATTATCGACGAGCGCCTGATCAACCCC
GACGGCTCCCTGCTGTTCCGAGTAACCATCAACGGAGTGACCGGCTGGCGGC
TGTGCGAACGCATTCTGGCGTAA

>ReNL
ATGGTGAGCAAGGGCGAGGAGGTCATCAAAGAGTTCATGCGCTTCAAGGTG
CGCATGGAGGGCTCCATGAACGGCCACGAGTTCGAGATCGAGGGCGAGGGC
GAGGGCCGCCCCTACGAGGGCACCCAGACCGCCAAGCTGAAGGTGACCAAG
GGCGGCCCCCTGCCCTTCGCCTGGGACATCCTGTCCCCCCAGTTCATGTACG
GCTCCAAGGCGTACGTGAAGCACCCCGCCGACATCCCCGATTACAAGAAGCT
GTCCTTCCCCGAGGGCTTCAAGTGGGAGCGCGTGATGAACTTCGAGGACGG
CGGTCTGGTGACCGTGACCCAGGACTCCTCCCTGCAGGACGGCACGCTGATC
TACAAGGTGAAGATGCGCGGCACCAACTTCCCCCCCGACGGCCCCGTAATGC
AGAAGAAGACCATGGGCTGGGAGGCCTCCACCGAGCGCCTGTACCCCCGCG
ACGGCGTGCTGAAGGGCGAGATCCACCAGGCCCTGAAGCTGAAGGACGGCG
GCCACTACCTGGTGGAGTTCAAGACCATCTACATGGCCAAGAAGCCCGTGCA
ACTGCCCGGCTACTACTACGTGGACACCAAGCTGGACATCACCTCCCACAAC
GAGGACTACACCATCGTGGAACAGTACGAGCGCTCCGAGGGCCGCCACCAC
CTGTTCCTGGGGCATGGCACCGGCAGCACCGGCAGCGGCAGCTCCGGCACC
GCCTCCTCCGAGGACAACAACATGGCCGTCATCAAAGAGTTCATGCGCTTCA
AGGTGCGCATGGAGGGCTCCATGAACGGCCACGAGTTCGAGATCGAGGGCG
AGGGCGAGGGCCGCCCCTACGAGGGCACCCAGACCGCCAAGCTGAAGGTG
ACCAAGGGCGGCCCCCTGCCCTTCGCCTGGGACATCCTGTCCCCCCAGTTCA
TGTACGGCTCCAAGGCGTACGTGAAGCACCCCGCCGACATCCCCGATTACAA
GAAGCTGTCCTTCCCCGAGGGCTTCAAGTGGGAGCGCGTGATGAACTTCGA
GGACGGCGGTCTGGTGACCGTGACCCAGGACTCCTCCCTGCAGGACGGCAC
GCTGATCTACAAGGTGAAGATGCGCGGCACCAACTTCCCCCCCGACGGCCCC
GTAATGCAGAAGAAGACCATGGGCTGGGAGGCCTCCACCGAGCGCCTGTAC
CCCCGCGACGGCGTGCTGAAGGGCGAGATCCACCAGGCCCTGAAGCTGAAG
GACGGCGGCCACTACCTGGTGGAGTTCAAGACCATCTACATGGCCAAGAAG
CCCGTGCAACTGCCCGGCTACTACTACGTGGACACCAAGCTGGACATCACCT
CCCACAACGAGGACTACACCATCGTGGAACAGTACGAGCGCTCCGAGGGCC
GCCACCACCTGTTCCGGCTGGAAGATTTCGTTGGGGACTGGCGACAGACAG
CCGGCTACAACCTGGACCAAGTCCTTGAACAGGGAGGTGTGTCCAGTTTGTT
TCAGAATCTCGGGGTGTCCGTAACTCCGATCCAAAGGATTGTCCTGAGCGGT
GAAAATGGGCTGAAGATCGACATCCATGTCATCATCCCGTATGAAGGTCTGA
GCGGCGACCAAATGGGCCAGATCGAAAAAATTTTTAAGGTGGTGTACCCTGT
GGATGATCATCACTTTAAGGTGATCCTGCACTATGGCACACTGGTAATCGACG
GGGTTACGCCGAACATGATCGACTATTTCGGACGGCCGTATGAAGGCATCGC
CGTGTTCGACGGCAAAAAGATCACTGTAACAGGGACCCTGTGGAACGGCAA
CAAAATTATCGACGAGCGCCTGATCAACCCCGACGGCTCCCTGCTGTTCCGA
GTAACCATCAACGGAGTGACCGGCTGGCGGCTGTGCGAACGCATTCTGGCGT
AA

>GeNL(Ca^{2+})_520
ATGGTGTCCAAGGGCGAAGAGGACAACATGGCCAGCCTGCCTGCCACCCAC
GAGCTGCACATCTTCGGCAGCATCAACGGCGTGGACTTCGACATGGTGGGAC
AGGGCACCGGCAACCCCAACGACGGCTACGAGGAACTGAACCTGAAGTCCA
CAAAGGGCGACCTGCAGTTCAGCCCCTGGATTCTGGTGCCCCACATCGGCTA
CGGCTTCCACCAGTACCTGCCCTACCCCGACGGCATGAGCCCTTTCCAGGCC
GCTATGGTGGATGGCAGCGGCTACCAGGTGCACCGGACCATGCAGTTTGAGG
ACGGCGCCAGCCTGACCGTGAACTACCGGTACACATACGAGGGCAGCCACAT

CAAGGGCGAGGCCCAAGTGAAGGGCACAGGCTTTCCAGCCGACGGCCCCGT
GATGACCAATAGCCTGACAGCCGCCGACTGGTGCAGAAGCAAGAAAACCTA
CCCCAATGACAAGACCATCATCAGCACCTTCAAGTGGTCCTACACCACCGGC
AATGGCAAGCGGTACAGAAGCACCGCCCGGACCACCTACACCTTCGCCAAA
CCTATGGCCGCCAACTACCTGAAGAACCAGCCTATGTACGTGTTCCGCAAGA
CCGAGCTGAAGCACTCCAAGACAGAACTGAACTTCAAAGAGTGGCAGAAA
GCCTTCACCGGGTTTGAAGATTTCGTTGGGGACTGGCGACAGACAGCCGGCT
ACAACCTGGACCAAGTCCTTGAACAGGGAGGTGTGTCCAGTTTGTTTCAGAA
TCTCGGGGTGTCCGTAACTCCGATCCAAAGGATTGTCCTGAGCGGTGAAAAT
GGGCTGAAGATCGACATCCATGTCATCATCCCGTATGAAGGTCCATGGATGCA
TGACCAACTGACAGAAGAGCAGATTGCAGAGTTCAAAGAAGCCTTCTCATTA
TTCGACAAGGATGGGGACGGCACCATCACCACAAGGGAACTTGGCACCGTT
ATGAGGTCGCTTGGACAAAACCCAACGGAAGCAGAATTGCAGGATATGATCA
ATGAAGTCGATGCTGATGGCAATGGAACGATTTACTTTCCTGAATTTCTTACTA
TGATGGCTAGAAAAATGAAGGACACAGACAGCGAAGAGGAAATCCGAGAA
GCATTCCGTGTTTTTGACAAGGATGGGAACGGCTACATCAGCGCTGCTCAGT
TACGTCACGTCATGACAAACCTCGGGGTGAAGTTAACAGATGAAGAAGTTGA
TGAAATGATAAGGGAAGCAGATATCGATGGTGATGGCCAAGTAAACTATGAA
GAGTTTGAACAAATGATGACAGCAAAGGGGGGGAAGAGGCGCTGGAAGAA
AAACTTCATTGCCGTCAGCGCTGCCAACCGGTTCAAGAAGATCTCCAGCTCC
GGGGCACTGGAGCTCCTGAGCGGCGACCAAATGGGCCAGATCGAAAAAATT
TTTAAGGTGGTGTACCCTGTGGATGATCATCACTTTAAGGTGATCCTGCACTA
TGGCACACTGGTAATCGACGGGGTTACGCCGAACATGATCGACTATTTCGGA
CGGCCGTATGAAGGCATCGCCGTGTTCGACGGCAAAAAGATCACTGTAACAG
GGACCCTGTGGAACGGCAACAAAATTATCGACGAGCGCCTGATCAACCCCG
ACGGCTCCCTGCTGTTCCGAGTAACCATCAACGGAGTGACCGGCTGGCGGCT
GTGCGAACGCATTCTGGCGTAA

>GeNL(Ca^{2+})_480
ATGGTGTCCAAGGGCGAAGAGGACAACATGGCCAGCCTGCCTGCCACCCAC
GAGCTGCACATCTTCGGCAGCATCAACGGCGTGGACTTCGACATGGTGGGAC
AGGGCACCGGCAACCCCAACGACGGCTACGAGGAACTGAACCTGAAGTCCA
CAAAGGGCGACCTGCAGTTCAGCCCCTGGATTCTGGTGCCCCACATCGGCTA
CGGCTTCCACCAGTACCTGCCCTACCCCGACGGCATGAGCCCTTTCCAGGCC
GCTATGGTGGATGGCAGCGGCTACCAGGTGCACCGGACCATGCAGTTTGAGG
ACGGCGCCAGCCTGACCGTGAACTACCGGTACACATACGAGGGCAGCCACAT
CAAGGGCGAGGCCCAAGTGAAGGGCACAGGCTTTCCAGCCGACGGCCCCGT
GATGACCAATAGCCTGACAGCCGCCGACTGGTGCAGAAGCAAGAAAACCTA
CCCCAATGACAAGACCATCATCAGCACCTTCAAGTGGTCCTACACCACCGGC
AATGGCAAGCGGTACAGAAGCACCGCCCGGACCACCTACACCTTCGCCAAA
CCTATGGCCGCCAACTACCTGAAGAACCAGCCTATGTACGTGTTCCGCAAGA
CCGAGCTGAAGCACTCCAAGACAGAACTGAACTTCAAAGAGTGGCAGAAA
GCCTTCACCGGGTTTGAAGATTTCGTTGGGGACTGGCGACAGACAGCCGGCT
ACAACCTGGACCAAGTCCTTGAACAGGGAGGTGTGTCCAGTTTGTTTCAGAA
TCTCGGGGTGTCCGTAACTCCGATCCAAAGGATTGTCCTGAGCGGTGAAAAT
GGGCTGAAGATCGACATCCATGTCATCATCCCGTATGAAGGTCCATGGATGCA
TGACCAACTGACAGAAGAGCAGATTGCAGAGTTCAAAGAAGCCTTCTCATTA
TTCGACAAGGATGGGGACGGCACCATCACCACAAGGGAACTTGGCACCGTT
ATGAGGTCGCTTGGACAAAACCCAACGGAAGCAGAATTGCAGGATATGATCA
ATGAAGTCGATGCTGATGGCAATGGAACGATTTACTTTCCTGAATTTCTTACTA
TGATGGCTAGAAAAATGAAGGACACAGACAGCGAAGAGGAAATCCGAGAA
GCATTCCGTGTTTTTGACAAGGATGGGAACGGCTACATCAGCGCTGCTCAGT
TACGTCACGTCATGACAAACCTCGGGGTGAAGTTAACAGATGAAGAAGTTGA
TGAAATGATAAGGGAAGCAGATATCGATGGTGATGGCCAAGTAAACTATGAA

GAGTTTGAACAAATGATGACAGCAAAGGGTGGCTCCAAGAGGCGCTGGAAG
AAAAACTTCATTGCCGTCAGCGCTGCCAACCGGTTCAAGAAGATCTCCAGCT
CCGGGGCACTGGAGCTCCTGAGCGGCGACCAAATGGGCCAGATCGAAAAAA
TTTTTAAGGTGGTGTACCCTGTGGATGATCATCACTTTAAGGTGATCCTGCAC
TATGGCACACTGGTAATCGACGGGGTTACGCCGAACATGATCGACTATTTCGG
ACGGCCGTATGAAGGCATCGCCGTGTTCGACGGCAAAAAGATCACTGTAACA
GGGACCCTGTGGAACGGCAACAAAATTATCGACGAGCGCCTGATCAACCCC
GACGGCTCCCTGCTGTTCCGAGTAACCATCAACGGAGTGACCGGCTGGCGGC
TGTGCGAACGCATTCTGGCGTAA

>GeNL(Ca^{2+})_250
ATGGTGTCCAAGGGCGAAGAGGACAACATGGCCAGCCTGCCTGCCACCCAC
GAGCTGCACATCTTCGGCAGCATCAACGGCGTGGACTTCGACATGGTGGGAC
AGGGCACCGGCAACCCCAACGACGGCTACGAGGAACTGAACCTGAAGTCCA
CAAAGGGCGACCTGCAGTTCAGCCCCTGGATTCTGGTGCCCCACATCGGCTA
CGGCTTCCACCAGTACCTGCCCTACCCCGACGGCATGAGCCCTTTCCAGGCC
GCTATGGTGGATGGCAGCGGCTACCAGGTGCACCGGACCATGCAGTTTGAGG
ACGGCGCCAGCCTGACCGTGAACTACCGGTACACATACGAGGGCAGCCACAT
CAAGGGCGAGGCCCAAGTGAAGGGCACAGGCTTTCCAGCCGACGGCCCCGT
GATGACCAATAGCCTGACAGCCGCCGACTGGTGCAGAAGCAAGAAAACCTA
CCCCAATGACAAGACCATCATCAGCACCTTCAAGTGGTCCTACACCACCGGC
AATGGCAAGCGGTACAGAAGCACCGCCCGGACCACCTACACCTTCGCCAAA
CCTATGGCCGCCAACTACCTGAAGAACCAGCCTATGTACGTGTTCCGCAAGA
CCGAGCTGAAGCACTCCAAGACAGAACTGAACTTCAAAGAGTGGCAGAAA
GCCTTCACCGGGTTTGAAGATTTCGTTGGGGACTGGCGACAGACAGCCGGCT
ACAACCTGGACCAAGTCCTTGAACAGGGAGGTGTGTCCAGTTTGTTTCAGAA
TCTCGGGGTGTCCGTAACTCCGATCCAAAGGATTGTCCTGAGCGGTGAAAAT
GGGCTGAAGATCGACATCCATGTCATCATCCCGTATGAAGGTCCATGGATGCA
TGACCAACTGACAGAAGAGCAGATTGCAGAGTTCAAAGAAGCCTTCTCATTA
TTCGACAAGGATGGGGACGGCACCATCACCACAAGGGAACTTGGCACCGTT
ATGAGGTCGCTTGGACAAAACCCAACGGAAGCAGAATTGCAGGATATGATCA
ATGAAGTCGATGCTGATGGCAATGGAACGATTTACTTTCCTGAATTTCTTACTA
TGATGGCTAGAAAAATGAAGGACACAGACAGCGAAGAGGAAATCCGAGAA
GCATTCCGTGTTTTTGACAAGGATGGGAACGGCTACATCAGCGCTGCTCAGT
TACGTCACGTCATGACAAACCTCGGGGTGAAGTTAACAGATGAAGAAGTTGA
TGAAATGATAAGGGAAGCAGATATCGATGGTGATGGCCAAGTAAACTATGAA
GAGTTTGAACAAATGATGACAGCAAAGGGAGGTGGCTCCAAGAGGCGCTGG
AAGAAAAACTTCATTGCCGTCAGCGCTGCCAACCGGTTCAAGAAGATCTCCA
GCTCCGGGGCACTGGAGCTCCTGAGCGGCGACCAAATGGGCCAGATCGAAA
AAATTTTTAAGGTGGTGTACCCTGTGGATGATCATCACTTTAAGGTGATCCTG
CACTATGGCACACTGGTAATCGACGGGGTTACGCCGAACATGATCGACTATTT
CGGACGGCCGTATGAAGGCATCGCCGTGTTCGACGGCAAAAAGATCACTGTA
ACAGGGACCCTGTGGAACGGCAACAAAATTATCGACGAGCGCCTGATCAAC
CCCGACGGCTCCCTGCTGTTCCGAGTAACCATCAACGGAGTGACCGGCTGGC
GGCTGTGCGAACGCATTCTGGCGTAA

>GeNL(Ca^{2+})_60
ATGGTGTCCAAGGGCGAAGAGGACAACATGGCCAGCCTGCCTGCCACCCAC
GAGCTGCACATCTTCGGCAGCATCAACGGCGTGGACTTCGACATGGTGGGAC
AGGGCACCGGCAACCCCAACGACGGCTACGAGGAACTGAACCTGAAGTCCA
CAAAGGGCGACCTGCAGTTCAGCCCCTGGATTCTGGTGCCCCACATCGGCTA
CGGCTTCCACCAGTACCTGCCCTACCCCGACGGCATGAGCCCTTTCCAGGCC
GCTATGGTGGATGGCAGCGGCTACCAGGTGCACCGGACCATGCAGTTTGAGG
ACGGCGCCAGCCTGACCGTGAACTACCGGTACACATACGAGGGCAGCCACAT

CAAGGGCGAGGCCCAAGTGAAGGGCACAGGCTTTCCAGCCGACGGCCCCGT
GATGACCAATAGCCTGACAGCCGCCGACTGGTGCAGAAGCAAGAAAACCTA
CCCCAATGACAAGACCATCATCAGCACCTTCAAGTGGTCCTACACCACCGGC
AATGGCAAGCGGTACAGAAGCACCGCCCGGACCACCTACACCTTCGCCAAA
CCTATGGCCGCCAACTACCTGAAGAACCAGCCTATGTACGTGTTCCGCAAGA
CCGAGCTGAAGCACTCCAAGACAGAACTGAACTTCAAAGAGTGGCAGAAA
GCCTTCACCGGGTTTGAAGATTTCGTTGGGGACTGGCGACAGACAGCCGGCT
ACAACCTGGACCAAGTCCTTGAACAGGGAGGTGTGTCCAGTTTGTTTCAGAA
TCTCGGGGTGTCCGTAACTCCGATCCAAAGGATTGTCCTGAGCGGTGAAAAT
GGGCTGAAGATCGACATCCATGTCATCATCCCGTATGAAGGTCCATGGATGCA
TGACCAACTGACAGAAGAGCAGATTGCAGAGTTCAAAGAAGCCTTCTCATTA
TTCGACAAGGATGGGGACGGCACCATCACCACAAGGGAACTTGGCACCGTT
ATGAGGTCGCTTGGACAAAACCCAACGGAAGCAGAATTGCAGGATATGATCA
ATGAAGTCGATGCTGATGGCAATGGAACGATTTACTTTCCTGAATTTCTTACTA
TGATGGCTAGAAAAATGAAGGACACAGACAGCGAAGAGGAAATCCGAGAA
GCATTCCGTGTTTTTGACAAGGATGGGAACGGCTACATCAGCGCTGCTGAAT
TACGTCACGTCATGACAAACCTCGGGGTGAAGTTAACAGATGAAGAAGTTGA
TGAAATGATAAGGGAAGCAGATATCGATGGTGATGGCCAAGTAAACTATGAA
GAGTTTGAACAAATGATGACAGCAAAGGGGGGAGGTGGCTCCAAGAGGCGC
TGGAAGAAAAACTTCATTGCCGTCAGCGCTGCCAACCGGTTCAAGAAGATCT
CCAGCTCCGGGGCACTGGAGCTCCTGAGCGGCGACCAAATGGGCCAGATCG
AAAAAATTTTTAAGGTGGTGTACCCTGTGGATGATCATCACTTTAAGGTGATC
CTGCACTATGGCACACTGGTAATCGACGGGGTTACGCCGAACATGATCGACTA
TTTCGGACGGCCGTATGAAGGCATCGCCGTGTTCGACGGCAAAAAGATCACT
GTAACAGGGACCCTGTGGAACGGCAACAAAATTATCGACGAGCGCCTGATC
AACCCCGACGGCTCCCTGCTGTTCCGAGTAACCATCAACGGAGTGACCGGCT
GGCGGCTGTGCGAACGCATTCTGGCGTAA

Supplementary References

1. Leutenegger, M. & Lasser, T. Detection efficiency in total internal reflection fluorescence microscopy. *Opt Express* **16**, 8519-8531 (2008).
2. Watanabe, R. et al. Biased Brownian stepping rotation of FoF1-ATP synthase driven by proton motive force. *Nat Commun* **4**, 1631 (2013).
3. Li, Y., Sierra, A.M., Ai, H.W. & Campbell, R.E. Identification of sites within a monomeric red fluorescent protein that tolerate peptide insertion and testing of corresponding circular permutations. *Photochem Photobiol* **84**, 111-119 (2008).
